# Editorial: Legume breeding in transition: innovation and outlook

**DOI:** 10.3389/fgene.2023.1221551

**Published:** 2023-05-30

**Authors:** Aamir Raina, Rafiul Amin Laskar, Samiullah Khan, Nasya Borisova Tomlekova, Waltram Ravelombola, Mahendar Thudi

**Affiliations:** ^1^ Mutation Breeding Laboratory, Department of Botany, Aligarh Muslim University, Aligarh, India; ^2^ Botany Section, Women’s College, Aligarh Muslim University, Aligarh, India; ^3^ Department of Botany, Bahona College, Jorhat, India; ^4^ Maritsa Vegetable Crops Research Institute, Plovdiv, Bulgaria; ^5^ Texas A&M University, College Station, TX, United States; ^6^ Dr. Rajendra Prasad Central Agricultural University, Samastipur, India

**Keywords:** legumes (Fabaceae), food security and nutrition, biotic and abiotic stress, underutilized legumes/orphan legumes, QTLs (quantitative trait loci), GWAS (genome-wide association study), MAS (marker assisted selection), transcriptome analysis

## Introduction

Legumes are important crops that are primarily harvested for their grains, which are rich in proteins, minerals, and other nutrients such as vitamins, fibre, and antioxidants. Legumes are mostly self-pollinated crops, which means they have a narrow genetic base, that poses challenges to crop improvement programs. Still, conventional and modern breeding approaches have contributed significantly to improving the agronomic traits, stress tolerance, and nutritional qualities of legume crops. Conventional breeding involves exposing plant propagules to mutagens and/or crossing two or more plants to generate a new generation with desired traits, while modern breeding approaches include molecular breeding, marker-assisted selection, and genetic engineering techniques. Through these approaches, researchers have been able to develop legume varieties with improved yield, disease resistance, drought tolerance, and nutritional qualities such as higher protein content, iron, zinc, and other essential micronutrients. Both conventional modern breeding approaches have achieved much success in cereal crops and very little attention has been given toward the improvement of legume crops. The genetic improvement of major and underutilized legume crops remains a major challenge in the path leading to the goal of global food security and nutrition. This Research Topic hosted at Frontiers in Genetics entitled “Legume Breeding in Transition: Innovation and Outlook” presents a series of research articles and reviews covering new understandings in the areas of germplasm diversity, transcriptomics, sequencing, genomics, marker assisted backcross breeding, genome wide association study, genome editing, machine learning algorithms and agronomy integrating theoretical, and experimental approaches.

## What have we achieved so far in legume breeding?

Recent advances in genomics offer hope for future genetic improvements in important legumes. Completing genome assemblies and resequencing efforts of large germplasm collections have made it possible to identify the underlying genes governing various important traits, which can enhance genetic gain and help develop more climate-resilient cultivars. Adzuki bean, cluster bean, horse Gram, lathyrus, red clover, urd bean, and winged bean are among the underutilized legumes that can benefit from these advancements in genomics. Gayacharan et al., reported that genetic gain in legumes can be enhanced by mining approximately 0.4 million *ex-situ* collections of legumes in gene banks. This would facilitate the identification of ideal donors for various agronomic traits. Jha et al., highlighted the significant advances made in developing genomic resources for underused legumes. These included genome-wide molecular markers, genome sequencing, genetic linkage maps, and trait mapping played a vital role in increasing legume production, which can contribute to global food security. Integrating genomic resources with unique breeding expertise and good seed system techniques can help increase the production of underused legumes and contribute to global food security.

In legume breeding, yield stability and adaptability are of prime importance, which requires yield trials at different geographic conditions to assess the impact of environmental factors. Pobkhunthod et al., identified KUP12BS029-1-1-3 large-seeded peanut genotype with significantly stable yield potential through multilocation yield trial using GGE biplot analysis in Thailand.

The seed size and shape are directly correlated to the overall quantity and quality of the lentil production. By controlling cell division via cell expansion and overall seed growth, Dutta et al., using a transcriptomic approach, demonstrated how essential genes, including kinases, transcription factors, cell wall-building enzymes, and hormone production pathways, are involved in lentil seed size regulation.

Bhat et al., identified 57 significant SNPs and six stable QTL regions using GWAS and haplotype alleles for improvement of yield and yield-related traits in soybean. In legume breeding, plant traits and soil attributes also play a role in achieving the desired production goals. While working on 797 soybean lines they indicated that the availability of soil texture information prior to the growing season might maximize the efficiency of a breeding program by allowing the reconsideration of experimental field design, allocation of resources, reduction of preliminary trials, and shortening of the breeding cycle (Vieira et al.).

## Biotic and abiotic stresses are the main obstacles in achieving the desired goals of legume production

Legumes are susceptible to biotic stresses that can adversely affect their growth and production, leading to significant yield losses. Biotic stresses include diseases caused by pathogens such as fungi, bacteria, viruses, and nematodes, as well as pests like insects and mites. Kaur et al., introgressed cry1Ac from transgenic chickpea lines into commercial cultivars using Marker-Assisted Backcrossing (MABC) breeding for pod borer resistance, which caused 100% *H. armigera* larval mortality. Using MABC, Bharadwaj et al., have developed a high yielding *Fusarium* wilt resistant chickpea cultivar BGM20211 by gene pyramiding and released it as Pusa Manav/Pusa Chickpea 20211 for commercial cultivation. One of the most common diseases that severely influence soybean output worldwide is *Phytophthora* rot and stem rot (PRSR) caused by *Phytophthora sojae*. Chandra et al., highlighted the developments made in understanding the genetic basis of PRSR resistance, genomic developments, and prospective uses of PRSR resistance in soybean for long-term control. Another important pulse crop, peanut is affected by many soil borne diseases and pathogens and substantially reduces yield. Sharif et al., identified pericarp abundant promotor AhGLP17-1P. Such promotors could drive the expression of defence-related genes in the pericarp and improve disease tolerance. Bacterial wilt is one of the primary diseases that cause a substantial decline in the common bean production. Zia et al., evaluated 168 accessions for resistance against bacterial wilt and identified 14 single nucleotide polymorphism (SNP) markers associated with the bacterial wilt resistance that can be utilized in developing new ideotypes of common bean with improved tolerance to bacterial wilt. Agarwal et al., identified a major QTL, “qpsd4-1,” on LG 4 and a minor QTL, “qpsd8-1,” on LG8 that explained 41.8% and 4.5% of phenotypic variance, respectively, associated with resistance to *Pythium ultimum* in chickpea.

Besides biotic stress, legumes are susceptible to various abiotic stresses such as drought, salinity, heat, cold, and heavy metal toxicity that significantly impact their production. Yin et al., identified 72 basic leucine zipper (bZIP) family transcription factors in the adzuki bean and concluded that tissue-specific bZIP might play a role in conferring tolerance to abiotic stress such as drought, cold, salt, and heavy metal stress. Frost is an important abiotic stress that reduces production, destroys nitrogen-fixing bacteria, and reduces diet value of legumes. Sallam et al., evaluated 185 genotypes of winter faba bean for frost tolerance and identified two frost tolerant genotypes *viz.*, S_028 and S_220. Using KASP markers and GWAS genotypes two markers *viz.*, Vf_Mt1g072640_001 and Vf_Mt7g073970_001 showed pleiotropic effects on root fresh and dry mass in both the genotypes. The two markers can be used for isolating frost tolerant genotypes and the two genotypes may be used as a donor of alleles for improving frost tolerance. Besides frost, floods also cause a substantial reduction in agricultural production. Floods are more detrimental to legumes, especially in pigeon pea, causing substantial crop loss. Using *De-novo* transcriptome assembly Tyagi et al., tried to decode the flood tolerance and identify the candidate genes that could help develop climate-resilient pigeon pea genotypes. Thus, effectively managing abiotic stresses is crucial for sustainable legume production and food security.

## Omics approaches in improving the nutritional profile and agronomic traits of legumes

Micronutrients are essential for human and plant growth and development, and legumes are a rich source of many important micronutrients. Some of the crucial micronutrients found in legumes include iron (Fe), zinc (Zn), copper (Cu), manganese (Mn), magnesium (Mg), calcium (Ca), and molybdenum (Mo). Any essential micronutrient deficiency impairs the correct operation of cellular systems and has several metabolic and physiological ramifications. Nazir et al., identified 113 SNPs through GBS associated with most of the seed micronutrients on chromosome 3 and chromosome 11 in common bean, which showed significant phenotypic variance ranging from 13.50% to 21.74%. Baloch et al. reported that the DArT-3367607 marker on chromosome Pv03, among the six markers identified, showed the highest phenotypic variation (7.5%) with the significant association for seed Mg contents in Turkish common bean germplasm.

To address an escalating global food demand, it is essential to produce superior crop types with high yield, increased nutrition, disease, and insect resistance. In comparison to other crops, chickpea and other legume crops have much lower genetic gains due to their limited genetic base. We must quicken genetic gains—a cyclical process of finding new variations, applying selection, and fixing good traits—to fulfill future demand. Additionally, modern variations of *Cicer* must be infused with genetic diversity from landraces and wild *Cicer* species to sustain greater genetic gain for a longer period of time. Singh et al. reviewed the current status of the narrow chickpea genetic base and the scope of modern “Omics” technologies in breaking this bottleneck overcoming the yield limits and achieving higher genetic gains. Underutilized crops represent a treasure of genes that may be used to improve the agronomic traits of widely used legume crops. Verma et al., working on seed development of lesser-known pulse crops *viz.*, ricebean, reported 6,928 differentially expressed genes in bold and small-seeded genotypes and identified several genes for seed development related traits. Zhao et al., carried out a genome-wide association study (GWAS) of 178 peanut cultivars and reported several marker-trait associations and candidate genes associated with hundred seed weight, total number branches, and pod shape. High yielding genotypes are considered a promising way of achieving food security for a burgeoning population. Singh et al., evaluated a panel of 100 blackgram genotypes at two locations and identified 49 significant SNP associations representing 42 QTLs related to yield and yield attributing traits. Wang et al., while working on QTL analysis in soybean, reported genes associated with the regulation of symbiosis. The group reported 10 QTLs associated with type III effector NopAA in *Sinorhizobium fredii* HH103 which functions as glycosyl hydrolase and plays a critical role in nodulation. Such studies are important in gaining more insights into the underlying mechanism of nitrogen fixation, making nitrogen fixation, and alleviating the harmful effects of chemical fertilizers on human health and the environment. Kumari et al., evaluated 98 wild and cultivated *Vigna* accessions and identified marker-trait associations (MTAs) for traits such as days to first flowering, days to maturity plant height and hundred seed weight that may be utilized in *Vigna* improvement programs. These MTAs may also help gain insights into understanding the underlying mechanism controlling the expression of these traits in various *Vigna* species.

## Recent advancements in gene editing, genome sequencing, and machine learning algorithms in the field of legume breeding

Even though conventional breeding strategies have contributed in improving thousands of crop cultivars, however, these approaches are laborious and tedious and are not enough to deliver the improved products at the required pace to sustain a growing world population. However, the huge advancements made in all fields of science, including plant breeding overcame all the limitations of conventional breeding approaches. One such example is gene editing technology in legumes reviewed by (Baloglu et al.). This review article provides details of the comparative governmental regulatory restrictions on gene-edited crops in European Union and United States of America. Besides gene editing, artificial intelligence is an emerging technology for crop improvement in analyzing big data in phenomics and genomics. Aasim et al. reported using machine learning models for predicting and optimizing the tissue culture protocols in common bean, a recalcitrant crop. The main limitations of linkage mapping and positional cloning in mapping genomic loci controlling agronomic traits are their low resolution, low-throughput, and time requirement. With recent developments in genomics and sequencing techniques, Bulk segregant analysis sequencing (BSA-seq) and its related approaches, *viz.*, quantitative trait locus (QTL)-seq, bulk segregant RNA-Seq (BSR)-seq, and MutMap, helped breeders in rapid identification of genetic loci/QTLs controlling agronomic traits at high resolution, accuracy, reduced time span, and in a high-throughput manner. Majeed et al., reviewed the BSA-seq and its related approaches in crop breeding, along with their merits and demerits in trait mapping. Overall genomic tools such as molecular markers, gene editing, and transcriptomics can be utilized to accelerate the breeding process and improve the efficiency of crop improvement programs. In conclusion, the recent advancements in genomics provide an opportunity to improve important crop legumes and develop more climate-resilient, high-yielding, and nutritionally rich cultivars. These advancements can contribute significantly to global food security and help meet the increasing demand for high-quality food.

